# Synthesis and Characterization of Polymeric Microspheres Template for a Homogeneous and Porous Monolith

**DOI:** 10.3390/polym13213639

**Published:** 2021-10-22

**Authors:** Nur Faezah Ibadat, Clarence M. Ongkudon, Suryani Saallah, Mailin Misson

**Affiliations:** Bioprocess Engineering Research Groups, Biotechnology Research Institute, Universiti Malaysia Sabah, Jalan UMS, Kota Kinabalu 88400, Sabah, Malaysia; nurfaezahibadat@gmail.com (N.F.I.); clarence@ums.edu.my (C.M.O.); suryani@ums.edu.my (S.S.)

**Keywords:** monolith, templates, microsphere, polymer, homogeneous pore, surfactant

## Abstract

Monolith is an emerging technology applicable for separation, filtration, and chromatography due to its interconnected pore structure. However, the current templates used to form monolith pores are associated with poor heat dissipation, uneven pore size distribution, and relatively low mechanical strength during monolith scale-up. Templates made from polymeric microsphere particles were synthesized via a solvent evaporation technique using different types of polymer (polystyrene, polycaprolactone, polypropylene, polyethylene, and poly (vinyl-alcohol) at varied polymer (10–40 wt%) and surfactant (5–10%) concentrations. The resulting microsphere particles were tested as a monolith template for the formation of homogenous pores. Among the tested polymers, polystyrene at 10 wt% concentration demonstrated good particle morphology determined to around 1.94–3.45 µm. The addition of surfactant at a concentration of 7–10 wt% during microsphere synthesis resulted in the formation of well-shaped and non-aggregating microsphere particles. In addition, the template has contributed to the production of porous monoliths with enhanced thermal stability. The thermogravimetric analysis (TGA) indicated monolith degradation between 230 °C and 450 °C, implying the material excellent mechanical strength. The findings of the study provide insightful knowledge on the feasibility of polymeric microsphere particles as a pore-directing template to fabricate monoliths with desired pore structures.

## 1. Introduction

Monolith is defined as a single piece of a porous material made of macropores that allows substrate to flow through. The interest in the synthesis of porous monolith has been growing for various applications, including catalysis and adsorption [1], separation [2], energy storage [3], and sensing [4]. This is due to their intriguing properties of high surface area, chemical stability, and high pore volumes. Higher permeability and reduced flow resistance as a result of the interconnected pores enable high-speed separation providing high throughput, resolution, and separation in short run times [5,6]. It is important that the porosity of the monolith is well structured to enable mass transport and diffusion. With a large surface area and controllable hierarchical pore architecture, porous monolith has become the material of choice for the aforementioned industrial applications [7].

Monolith can be synthesized via free-radical polymerization and click reactions [5]. Polymethacrylate monoliths have also been prepared via free-radical polymerization as synthetic adsorbents with engineered macroporous and mesoporous interconnected channels [5]. The porous structure inside the monolith can be created via a template-free and templated method. Polymer phase separation is a template-free monolith synthesis that is both easy and cost-effective. Solvent plays a significant role in generating pores in synthetic media that can be readily removed from the polymeric framework [8]. Nevertheless, this method necessitates the use of two solvents, which may increase the toxicity and limit the biological applicability of monolith. A complicated phase separation in sol-gel reactions also has been known as a challenge in maintaining the porous structure in silica monoliths [9].

Meanwhile, a templated monolith is attractive due to its ability to fine-tune porosity. Templates are materials that serve as a pore-directing agent to create pores in a monolith. Common templates used in monolith fabrication are derived from biodegradable polymers and supramolecular aggregates [10]. Porogens such as hexane and polyethylene glycol are used as pore-forming agents in the current monolith [11]. The desired pore size, porosity, and pore morphology can be obtained by manipulating the porogen properties. However, this technique causes a drawback of poor pore interconnectivity as a result of incomplete porogen removal [12]. Furthermore, employing free-radical initiator results in random and closed pore architectures, which might lead to pressure build-up inside the reactor. Therefore, the search for appropriate templates capable of producing homogeneous and well-structured monolith pores presents a profound research interest.

Monolith templates can be categorized into hard and soft templates [13]. Soft templates include biological cells, virus particles, micro/miniemulsion droplets based on surfactant self-assemblies molecularly self-assembled structures such as micelles, vesicles, and other surfactant mesophases and liquid crystals. The hard template, on the other hand, consists of polymer microspheres, porous membrane, plastic foam, ion exchange resin, carbon fibre, and porous anodic aluminium oxide [14]. The hard template offers good control over the reaction condition and commonly deployed technique for the fabrication of nanostructured materials such as nanoparticles, nanorods, nanowire, and nanotubes. According to Ali [15], during the templating process, the first step is the filling or coating process of the pre-formed template with soft precursor material aiming to form the desired shape of the material. The precursor material is then fixed or hardens via a chemical or physical process before subsequently being removed after resembling the shape of the template.

Microspheres, also known as microparticles, are either microcapsule or monolithic particles containing small spherical particles with sizes ranging from 1 to 1000 μm [16,17,18]. There are three classes of pores that can be distinguished: micropores (linear dimension below 2 nm), mesopores (dimension 2–50 nm), and macropores (dimension above 50 nm) [19]. Polymeric microspheres offer good control of particle size and attract biotechnological interest due to their pore interconnectivity, low mass density, and high surface area [20]. Polymeric microspheres derived from various natural and synthetic polymers could be a potential candidate for monolith templates. Polymers are macromolecules derived by mixing one or more chemical units in a repetitive pattern characterized by its high relative molecular mass and formed an interconnecting large number of small molecules [21]. Synthetic polymers have advantages over natural polymers in terms of tunable characteristics and higher mechanical strength, allowing them to be used in a wider range of applications [22]. Microspheres made from polystyrene possess high surface areas and permeability [18], beneficial for various applications including in drug delivery, catalysis, separation, and chromatography system [20]. Simple method for the fabrication of well-ordered nanoporous materials from polystyrene-block-poly(dimethylsiloxane) (PS-PDMS) diblock copolymer has been developed [23]. Porous polymeric monoliths have also been prepared using poly(ethylene oxide)-*b*-polystyrene block copolymers stabilized high internal phase emulsion templates [24].

Polystyrene microspheres can be synthesized via spin coating, solvent evaporation, sedimentation, and self-assembly. The solvent evaporation technique has been recognized as a relatively simple technique feasible for an upscale production [25]. The desired particle sizes and interconnected channels can be engineered to target the hydrodynamic features and biotechnological applicability of the monolith. This can be achieved by fine-tuning the process conditions such as the polymer concentration, reaction temperature, and stirring speed [26]. Although numerous templates have been developed for porous monolith, polymeric microsphere particles have rarely been investigated as monolith templates. This work attempts to investigate the key synthesis process variables in preparing polymeric microspheres template. The effect of polymer concentration and surfactant concentration was investigated. The resulting microsphere-templated porous monolith was subsequently characterized.

## 2. Materials and Methods

### 2.1. Materials

Polystyrene, polycaprolactone, polyethylene, poly (vinyl-alcohol), polypropylene, dimethylformamide, xylene, ethylene glycol dimethacrylate (EDMA) 98%, glycidyl methacrylate (GMA) 97%, azobisisobutyronitrile (AIBN), and Brij O10 surfactant were all purchased from Sigma Aldrich (Milwaukee, WI, USA); toluene, dimethylformamide (DMF), and xylene were purchased from Fisher Chemical (Waltham, MA, USA); and polyethylene glycol (PEG) was purchased from Tokyo Chemical Industries (Tokyo, Japan).

### 2.2. Synthesis of Polymeric Microspheres Template

**Figure 1:**
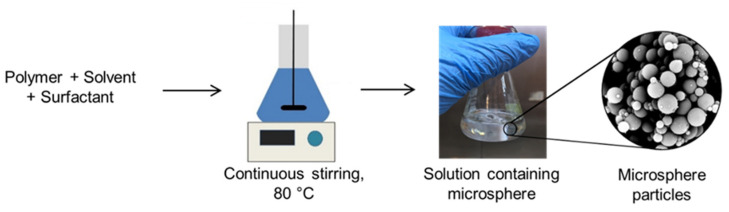
Schematic diagram of the synthesis of microspheres template via solvent evaporation method.

Microsphere templates were synthesized using a solvent evaporation method as described by [17] with slight modification. The schematic diagram illustrating the fabrication process of the microspheres template is shown in Figure 1. Three (3) parameters (effect of type of polymer, effect of polymer concentration and effect of surfactant concentration) were studied to produce an excellent quality of microsphere particles. Polystyrene, poly (vinyl-alcohol), polycaprolactone, polyethylene, and polypropylene polymers (1 g) were dissolved in 10 mL of their respective solvent (polystyrene in dimethylformamide, polycaprolactone in dimethylformamide, poly (vinyl-alcohol) in distilled water, polyethylene in xylene, and polypropylene in xylene) at 80 °C (Favorit, PLT Scientific Instruments, Kuala Lumpur, Malaysia) under continuous stirring at 1500 rpm (Favorit, PLT Scientific Instruments, Malaysia). The microsphere particles were synthesized at 80 °C following the method described by Esfandyari-Manesh et al. [27] with a high stirring rate (1500 rpm) to reduce the viscosity of polymer solution as suggested by Zhao et al. [28]. To investigate the effect of polymer concentration on the formation of microspheres, the polymer solutions were prepared at 10 wt%, 20 wt%, 30 wt%, and 40 wt% concentrations by dissolving the polymer beads about 1 g, 2 g, 3 g, and 4 g, correspondingly, inside 10 mL of their abovementioned respective solvent. Next, approximately 0.7 mL of Brij O10 surfactant was added into the solution as a stabilizer and stirred at 1500 rpm for 1 h at 80 °C [20]. As a result, the mixtures turned into transparent solutions. The reaction mixture was allowed to evaporate for 1 h. The resulting monolith samples were allowed to cool down at room temperature. Samples were kept closed and stored at room temperature until further use. The morphological structure and the diameter of the resultant particles were observed using scanning electron microscopy (SEM) and dynamic light scattering (DLS), respectively.

For the effect of surfactant concentration, approximately 5 wt% (0.5 mL), 7 wt% (0.7 mL), and 10 wt% (1 mL) of Brij O10 surfactant was added into the polymer solution and stirred at 1500 rpm for 1 h at 80 °C. Upon completion of the reaction, the solvent was allowed to evaporate. Samples were allowed to cool down at room temperature. The morphological structure and the diameter of the resultant particles were observed using SEM and DLS, respectively.

### 2.3. Synthesis of Microsphere-Templated Monolith

Hierarchically porous polymethacrylate monolith (PMMA) was synthesized via free-radical co-polymerization of functional monomer GMA and EDMA [29]. The monolith monomers were added with 1 wt% AIBN to initiate the polymerization reaction. Microsphere templates were added into the mixture at different template/monomers ratio (50:50, 60:40, and 70:30). Monoliths with a final volume of 20 mL were fabricated. The monomers and template mixtures were sonicated at 20 °C for 30 min, inserted into a casting mould and heated at 60 °C for 3 h inside a water bath. The resulting solid monolith was removed and kept at room temperature until further use. A monolith without the incorporation of templates was fabricated as a control study.

### 2.4. Removal of Microspheres Template from Monolith

The solvent treatment was employed by soaking the resultant solid monolith into toluene solvent overnight [30]. The monolith was washed with water to remove the solvents residues. Finally, the sample was oven dried at 60 °C and kept at room temperature until further use.

### 2.5. Characterization of Microspheres Template and Monoliths

The morphological observation of templates and microspheres was conducted using scanning electron microscopy (SEM) (Hitachi High Technologies America Inc. S-3400 N, Gaithersburg, MD, USA). Template or monolith samples were mounted on an SEM sampling holder using double-sided adhesive tape and coated with gold before the morphological examination was carried out. The average pore size of the polystyrene microsphere was observed under a dynamic light scattering (DLS) (Nanoplus Micromeritics Intrument Corp., Gerbrunn, Germany) instrument. The polymer solution was diluted with distilled water at a 1:1 ratio. Approximately 2 mL of the solution was transferred into a clean cuvette and placed into the column. The sample reading was repeated three (3) times. Fourier transform infrared (FTIR) (Agilent FTIR spectrometer, Santa Clara, CA, USA) was used to analyze the presence of specific functional groups in the monolith samples. The microsphere template and monolith samples (monolith with template and monolith after template removal) were placed in the sample holder. The FTIR spectra of the samples were recorded from 650 cm^−1^ to 3650 cm^−1^ wavelengths at room temperature. The thermal stability of monolith was analyzed using thermogravimetric analysis (TGA) (Mettler Toledo, Columbus, OH, USA). Approximately 20 mg of monolith sample was used for analysis in 70 μL aluminum oxide pans. The analysis was conducted at 10 °C/min over a temperature range of 50–700 °C and a nitrogen flow rate of 25 mL/min. The TGA system was frequently purged with nitrogen gas at a flow rate of 100 mL/min and a heat flow of 20 °C/min for equilibration. Isothermal degradation of the samples was measured at 150 °C for 120 min.

## 3. Results and Discussion

### 3.1. Synthesis of Polymeric Microsphere Particles

#### 3.1.1. Effect of Type of Polymer

**Figure 2:**
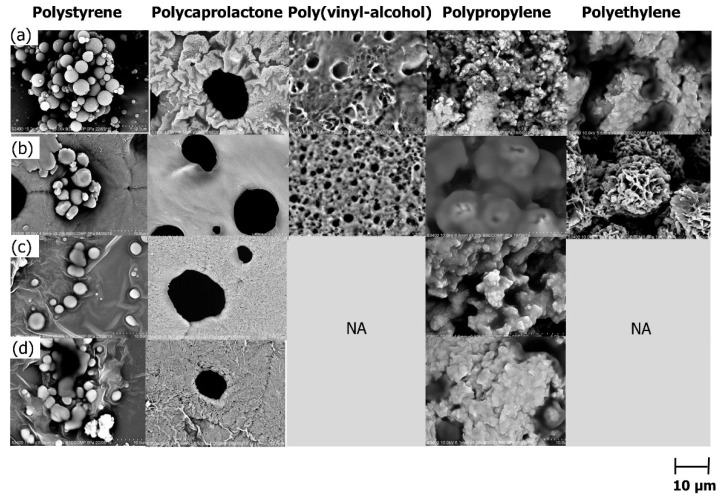
Polymeric templates from different types of polymer at different concentrations (**a**) 10 wt%, (**b**) 20 wt%, (**c**) 30 wt%, and (**d**) 40 wt%.

Polystyrene, polycaprolactone, poly (vinyl-alcohol), polyethylene, and polypropylene were tested as a chemical precursor for the synthesis of polymeric microspheres template. The polymer beads were dissolved in respective solvents. According to Miller-Chou and Koenig [31], the polymer dissolves upon exposure in a solvent involving solvent diffusion and chain disentanglement transport processes, creating an interconnected micro void. The SEM images of the resulting templates are shown in Figure 2. As can be seen, the type of polymer played a vital role for the formation of particles. The polystyrene observed successfully produced microsphere particles with a diameter around 2 µm to 10 µm. Polystyrene generated a good shape of microspheres while polycaprolactone and poly (vinyl-alcohol) demonstrated the formation of non-homogeneous pore-like structures. It was observed that the pores produced by poly (vinyl-alcohol) were bigger than the polycaprolactone. Microsponges or porous sponges also can be seen in the SEM images of poly (vinyl-alcohol). Microsponges are tiny sponge-like spherical particles with a large porous surface that are typically used in drug delivery systems [32].

Polyethylene and polypropylene, on the other hand, exhibited the formation of rough structures with some observable particle aggregations. Different polymers possessed different properties which largely affect the resulting microsphere’s morphological characteristics, and based on the data, it can be seen that different polymers caused diverse solution appearances. Only polystyrene produced good morphology at low concentrations; as the concentration increased, the morphology deteriorated. This is because when polymer concentration increases, solubility decreases. It may be concluded that among the tested polymers, polystyrene was found as the best polymer for microsphere synthesis via the solvent evaporation method.

#### 3.1.2. Effect of Polymer Concentration

The effect of polymer concentration ranging from 10 wt% to 40 wt% was further evaluated (Figure 2a–d). The polymers were dissolved in respective solvents.

The findings show that the polymer concentration influenced the morphological properties of the particles, as evident by the data by the polystyrene polymer (Figure 2a). The concentration at 10 wt% was found as the most optimal concentration to obtain good quality of microsphere particles using polystyrene. As the concentration increased, non-homogenous particles were observed while the highest concentration (40 wt%) demonstrated particles aggregation. At all tested concentrations, both polycaprolactone (Figure 2b) and poly (vinyl-alcohol) (Figure 2c) generated surfaces containing pores while polypropylene and polyethylene showed agglomerated-like particles. It is important to note that polypropylene and polyethylene did not dissolve in their respective solvents at concentrations of 30 wt% and 40 wt%, respectively. As a result, the polymer samples were not subjected to morphological examination, and no SEM image was acquired.

The findings show that the particle aggregation occurred in a high concentration of polymer which possesses a higher degree of viscosity. This phenomenon was also described by Johansen and Schæfer [33]. According to Shahzad et al. [34], particle aggregation increases the risks of channel clogging in monolithic columns and reduces the life span of the monoliths. Aggregation is a dynamic process influenced by both intramolecular and electrostatic forces which lead to the particle–particle interaction eventually forming a compact structure in a pattern [33]. Small aggregates form a bigger aggregate through agglomeration, and due to the presence of attractive interaction, the aggregates arrange themselves, forming a pattern called ‘channel bridging’.

#### 3.1.3. Effect of Surfactant Concentration

**Figure 3:**
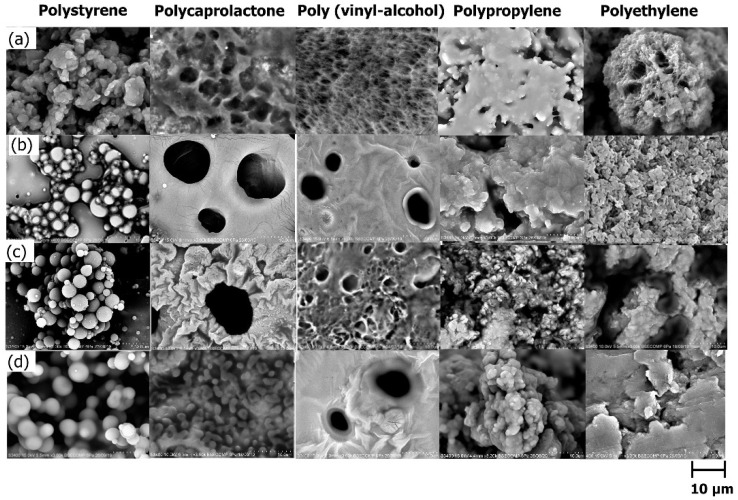
Polymeric templates using different concentrations of surfactant: (**a**) no addition of surfactant, (**b**) 5 wt%), (**c**) 7 wt%, and (**d**) 10 wt%.

The effect of surfactant concentration on the synthesis of microsphere particles was evaluated ranging from 5 wt% to 10 wt% of surfactants as presented in Figure 3. A control study was carried out without the addition of surfactant. It can be observed that microsphere particles were not detected at all tested polymers when no surfactant was added. However, as the concentration of surfactant increased, a better quality of particles was produced by polystyrene. The observation proved the role of surfactant as a particle stabilizer during monolith template synthesis as clarified by [20]. On the other hand, polyethylene and polypropylene consistently produced a rough structure with aggregation at all surfactant concentrations. Among the tested surfactant concentrations, 7 wt% and 10 wt% generated good morphology of the polystyrene microsphere particles. A low surfactant concentration is favorable considering the probable toxicity and enhanced cost the surfactant contributes at a higher concentration, which may lead to limited applications of the materials. Furthermore, a high surfactant concentration could lower the mechanical strength of the hollow materials as described by [35]. Hence, the surfactant concentration at 7 wt% was chosen for the template synthesis.

#### 3.1.4. Effect of Polymer and Surfactant Concentration on Particle Size

**Figure 4:**
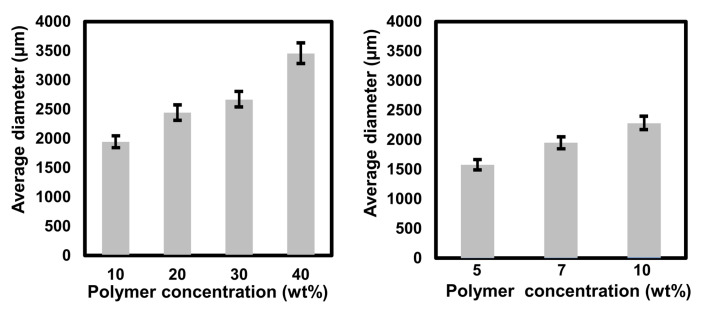
Particle diameter of polystyrene microsphere particles synthesized at different (**a**) polymer concentrations and (**b**) surfactant concentrations under continuous stirring rate at 1500 rpm at 80 °C (error bars shows standard deviation).

Figure 4a presents the average diameter of the microsphere particles at different concentrations of polystyrene. The particles diameter increased correspondingly with the concentration of polymer concentration. Polystyrene at 10 wt% concentration produced the smallest particle size determined at 1943.8 nm with the polydispersity index recorded at 0.537 while the highest polymer concentration yielded the biggest particle size (3454.9 nm) with the polydispersity index at 0.917. Higher PDI values are found in samples with a wider range of particle sizes, while lower PDI values are found in samples with evenly sized particles [36]. This increasing trend of the particle sizes as the concentration of polymer increased indicates that the polymer concentration might play an important role in determining the resultant microsphere particles. This phenomenon was probably due to high viscosity at a higher polymer concentration that caused a non-homogenous dispersion and subsequently led to the formation of larger particle diameter [37]. The effect of surfactant concentration on the average diameter of the particle is shown in Figure 4b. The data show a similar trend of increasing average pore diameter size as the surfactant concentration increased. Increasing the polymer concentration leads to a higher viscosity level in the solution, thus causing an increase in the emulsion droplet size [38]. It may be concluded that the concentrations of polymer and surfactant play an important part in determining the particle size of microspheres.

Based on the findings, polystyrene polymer at 10 wt% concentration was found to have successfully produced the desired microsphere particles, as evident by the SEM images. The addition of surfactant at 7–10 wt% as a particle stabilizer into polystyrene solution could generate non-aggregating microspheres. The polystyrene microspheres were further used as a template for the fabrication of porous monolith. The microsphere particles and the resulting microsphere-templated monolith were further characterized.

### 3.2. Synthesis of Microsphere-Templated Porous Monolith

**Figure 5:**
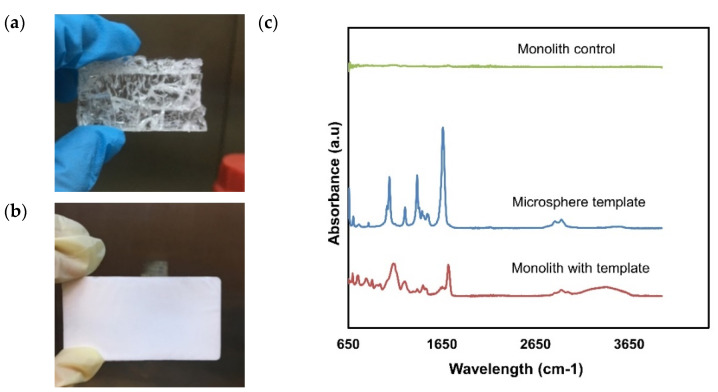
Physical observation of monolith (**a**) without template and (**b**) with template, and (**c**) FTIR spectra of microsphere templates and monoliths with and without template.

The microspheres template was further tested as a pore-directing agent in monolith synthesis. The general monolith fabrication procedure involves mixing the monomeric, crosslinker and initiator in favorable reaction conditions. The suspension of GMA, EDMA, AIBN, and microspheres template were homogenously mixed to allow polymerization to occur. The composition of the monomers and template in the initial polymerization mixture affect the resulting pores of the monolith. Upon the addition of the microspheres template into the mixture of GMA/EDMA (7:3 ratio) with 1% AIBN, the mixtures were sonicated for 30 min. It was observed that the suspension was homogenously dispersed and formed a white solid monolith resembling the casting mould after heating at 60 °C for 3 h. The resulting monolith was compared with monolith prepared without a template, as shown in Figure 5a,b. As can be seen that the non-templated monolith produced a crystal-like and transparent structure (Figure 5a). Meanwhile, the microsphere-templated monolith produced a solid white material, as Figure 5b indicates. The FTIR spectra of both monoliths (with and without template) were observed and compared with the FTIR spectra of the microspheres template. There were no obvious peaks observed in the FTIR spectra of monolith without template. The most probable reason could be due to weak interaction among the monomers and initiator during polymerization in the absence of pore-directing template, thus producing a non-desirable monolith, as shown in Figure 5a. Poly (GMA-co-EDMA) monolith typically possess epoxy and carbonyl functional groups. As shown in the FTIR spectra of polymethacrylate monolith with template, there were characteristic bands at 1730 cm^−1^, which were due to C=O stretch of the monolith. In addition, the bands at 2980 cm^−1^ corresponded to C–H stretching vibration [39].

No obvious peak was observed in the control monolith. Meanwhile, the microsphere-templated monolith (Figure 5c) demonstrated FTIR spectra that corresponded with the spectra of the microspheres template. The C–O and O–H stretching assigned at 1088 cm^−1^ and 2925 cm^−1^, respectively, demonstrated vibration of carbonyl and alcohol in the surfactant Brij O10 used as a particle stabilizer during polystyrene microsphere synthesis. This surfactant is non-ionic ethoxylated based on oleyl alcohol. The –C=C– and C–H stretching bands are represented by the pronounced peaks at 2856 cm^−1^ while the peak at 2925 cm^−1^ signifies the presence of alkane and alkene groups in dimethylformamide solvent of polystyrene. These peaks were found to be available in the FTIR spectra of the templated monolith. The FTIR analyses demonstrated that the microspheres template was successfully incorporated into the monolith structure.

### 3.3. Effect of Microsphere Template on Pore Formation

**Figure 6:**
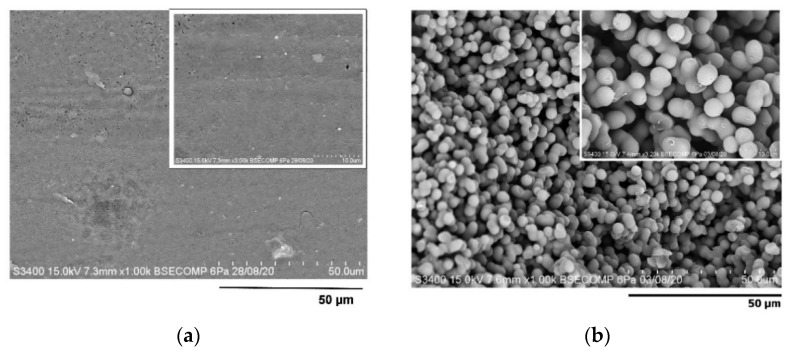
SEM images of monolith (**a**) without template and (**b**) with template.

The monolith was synthesized via free radical polymerization using the synthesized microsphere particles as a template-directing agent and compared with a non-templated monolith as control. The physicochemical properties of the templated and non-templated monoliths were further analyzed. Observation under SEM revealed a non-porous monolith was obtained in the monolith synthesized without template (Figure 6a). This is in parallel with no obvious peak formations as observed in FTIR spectra by the non-templated monolith (Figure 5c). Meanwhile, the microsphere-templated monolith demonstrated the formation of homogenous pore structures (Figure 6b). The findings show that the template is an important material that can create pore structure within monolith surfaces. The porosity of the monolith can be controlled by fine-tuning the particle size of the microspheres template.

### 3.4. Effect of Microspheres Template on Monolith Thermal Stability

**Figure 7:**
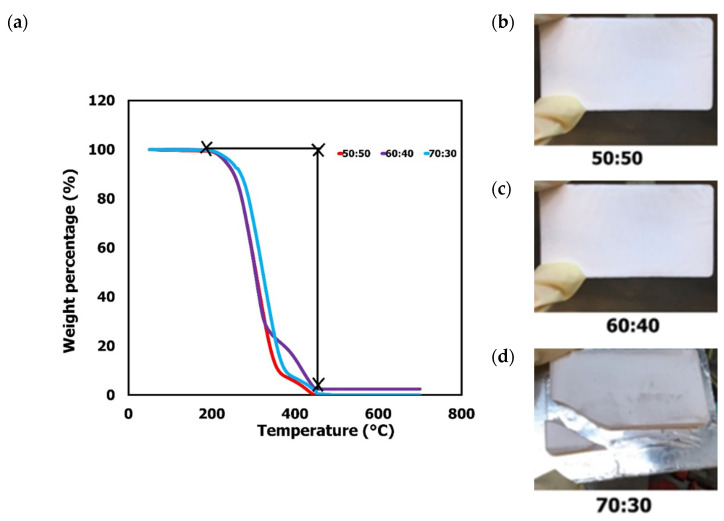
TGA analysis of microsphere-templated monolith at temperatures ranging from 50 °C to 700 °C (**a**) and the physical structure of the monoliths prepared at 50:50 (**b**), 60:40 (**c**), and 70:30 (**d**) ratio.

Thermogravimetric analysis (TGA) determines the thermal stability of material against an elevated temperature. In order to determine the effect of the microspheres template on monolith stability, monoliths at different template/monomer ratios were synthesized (50:50, 60:40, and 70:30). The TGA analysis of the respective monolith is presented in Figure 7a. The physical structure of the monoliths prepared at 50:50 (Figure 7b), 60:60 (Figure 7c), and 70:30 ratio (Figure 7d) was also observed. The first degradation of all monoliths was observed around 243 °C followed by the next degradation, which started to occur at 332 °C. The monolith samples were fully degraded and turned into ashes at 700 °C. In comparison with the monolith fabricated by Yusuf et al. [40], the first degradation was recorded at 210 °C, a slightly lower temperature that indicates greater thermal stability of this microsphere-templated monolith. Interestingly, the monolith stability is comparable and slightly improved the monolith prepared by Acquah et al. [5] using porogen as a pore-directing agent. The monolith, which was synthesized using similar operating conditions and precursors, demonstrated degradation around 200 °C. Moreover, it was found in this study that a suitable template ratio plays an important role in enhancing the thermal stability of the monolith. As can be seen, the monolith at 60:40 template/monomer ratio demonstrated an enhanced thermal degradation around 300 °C to 400 °C, in comparison with 50:50 and 70:30 ratio. Increasing the concentration of the template in monolith preparation lowered the physical strength of the resultant monolith, as evident by the fragile and brittle monolith in Figure 7d.

In summary, microsphere particles can be synthesized using polystyrene polymer at 10 wt% in the presence of surfactant as a particle stabilizer. The sizes of the microspheres obtained in this study (1.94–3.45 µm) are smaller than other reported studies on polymeric microspheres synthesized using the solvent evaporation method. The microspheres template successfully generated homogenous pores in monolith structure in comparison with the non-templated monolith. In addition, the template has contributed to the production of porous monolith with enhanced thermal stability.

## 4. Conclusions

A simple method to produce polymeric microsphere particles as a monolith template was demonstrated in this study. This study highlights the effects of variable parameters such as the type of polymer as a chemical precursor, concentration of polymer, and concentration of surfactant to stabilize the resulting microsphere particles. It is evident from this study that polystyrene at 10 wt% concentration plays a vital role in the formation of microsphere particles with good morphological characteristics. The addition of surfactant was found to stabilise the resulting particles, resulting in well-shaped and non-agglomerating microspheres. It has been revealed that the presence of the microspheres template has successfully produced solid and porous monoliths in comparison with its non-templated counterpart. In addition, an appropriate ratio of template/monomer led to monolith production with enhanced thermal stability up to 230–450 °C, as evidenced by the TGA analysis. The overall findings of this study demonstrate a great potential of polymeric microspheres as monolith templates, addressing the monolith challenges with closed and dead-end pores. Homogenous pore monoliths can be potentially applied in water filtration and bio-separation fields.

## Data Availability

Not applicable.

## Abbreviations

The following abbreviations are used in this manuscript:

TGA	Thermogravimetric analysis
nm	Nanometre
µm	Micrometre
EDMA	Ethylene glycol dimethacrylate
GMA	Glycidyl methacrylate
AIBN	Azobisisobutyronitrile
DMF	Dimethylformamide
SEM	Scanning Electron Microscopy
DLS	Dynamic light scattering
PMMA	Polymethacrylate monolith
FTIR	Fourier transform infrared
Rpm	Revolutions per minute
Mg	Milligram
Ml	Millilitre
Min	Minute
µL	Microlitre
(cm^−1^)	Wavelength Per unit distance
